# Elevated Levels of Lewis Y and Integrin α_5_β_1_ Correlate with Chemotherapeutic Drug Resistance in Epithelial Ovarian Carcinoma

**DOI:** 10.3390/ijms131215588

**Published:** 2012-11-23

**Authors:** Zhenhua Hu, Song Gao, Jian Gao, Rui Hou, Chuan Liu, Juanjuan Liu, Beibei Li, Dawo Liu, Shulan Zhang, Bei Lin

**Affiliations:** Department of Obstetrics and Gynecology, Shengjing Hospital affiliated to China Medical University, Shenyang 110004, Liaoning Province, China; E-Mails: huzh5062012@163.com (Z.H.); drgaosong@hotmail.com (S.G.); tbagd0519@sina.com (J.G.); hour@sj-hospital.org (R.H.); liuc1_88@yahoo.com.cn (C.L.); juanjuanliu_lg@yahoo.com.cn (J.L.); libeibeifeng@126.com (B.L.); cyldw2007@163.com (D.L.); zsl99@126.com (S.Z.)

**Keywords:** integrins, Lewis y antigen, ovarian caricinoma, chemoresistance

## Abstract

**Objective:**

To measure Lewis y and integrin α_5_β_1_ expression in epithelial ovarian carcinoma and to correlate the levels of these molecules with ovarian carcinoma chemotherapy and prognosis.

**Methods:**

The study population included 34 ovarian carcinoma patients with chemotherapeutic drug-resistance, six partially drug-sensitive cases, and 52 drug-sensitive cases (92 total). Immunochemistry was used to determine expression of Lewis y antigen and integrin α_5_β_1_ in ovarian carcinoma tissues, and correlation of these molecules with chemotherapy resistance was further investigated, Multi-factor logistic regression analysis was applied to investigate: age, surgical stage, grade, subtype of patient cases, metastasis of lymph nodes, residual tumor size, expression levels of Lewis y antigen and integrin α_5_β_1_ correlation with ovarian carcinoma chemotherapy resistance.

**Results:**

The expression rates of Lewis y antigen and integrins α_5_ and β_1_ were significantly greater in the drug-resistant group (91.17%, 85.29%, 88.24%) than the partially sensitive (50.00%, 33.33%, 50.00%) or sensitive groups (61.54%, 57.69%, 55.77%). Binary logistic regression analysis revealed that surgical stage, residual tumor size, and expression of integrin α_5_ and Lewis y in ovarian carcinoma tissues were independent risk factors for chemotherapeutic drug resistance.

**Conclusions:**

Overexpression of Lewis y and integrin α_5_ are strong risk factors for chemotherapeutic drug resistance in ovarian carcinoma patients.

## 1. Introduction

Because the onset of epithelial ovarian carcinoma is undetectable, most patients have already progressed to an advanced stage once they are accurately diagnosed. Treatment approaches typically involve surgical debulking of tumor cells and combined chemotherapy with carboplatin and paclitaxel, however intrinsic and acquired drug resistant responses can significantly affect treatment efficacy. It has been reported that approximately 75%–80% of patients respond to chemotherapy initially, with the remaining patients exhibiting primary drug resistance. Eventually, however, at least 80% of all chemotherapy patients manifest drug resistance phenomena [1]. There is a need for studies to investigate the drug resistance of ovarian carcinoma; the results from such studies may improve patient prognosis and enhance survival. Recently, cell adhesion mediated drug resistance (CAM-DR) has been implicated in tumor drug resistance [2–4]. It is well known that integrin, which mediates cell adhesion, produces CAM-DR by inhibiting apoptosis [5–7]. Integrin can be glycosylated, and this modification can enhance CAM-DR [7].

Integrin α_5_β_1_ is a transmembrane glycoprotein adhesion molecule that is expressed extensively on the cell surface and is organized as a dimer of α and β (type I) protein subunits. Integrin α_5_β_1_ is involved in signal transduction both between cells and between cells and the extracellular matrix. It plays a pivotal role in various physiological and pathological processes, including angiogenesis, tumor metastasis, and immune response. Several studies suggest that integrins α_5_ and β_1_ are overexpressed in ovarian carcinoma. Moreover, the expression intensities of these integrins are closely associated with malignant behavior in ovarian carcinoma [8,9].

The binding strengths between integrins and their ligands are controlled by a variety of factors, including glycosylation status. Our previous work demonstrated that the oligosaccharide chain, Lewis y, is present on the integrin α and β subunits [10,11]. Modification of α and β integrins by the addition of Lewis y antigen increased proliferation, reduced apoptosis, and increased chemoresistance [10]. In the present study, we measured the expression levels of Lewis y antigen and integrins α_5_ and β_1_. We evaluated their localization in ovarian carcinoma tissues obtained from drug-resistant and non-drug-resistant patients. The aim of this work was to analyze the relationships of Lewis y and integrin α_5_/β_1_ with ovarian carcinoma chemotherapeutic drug resistance and the outcome of the patients.

## 2. Results

### 2.1. Expression of Lewis y Antigen and Integrins α5 and β1 in Ovarian Carcinoma Tissues

**Figure 1:**
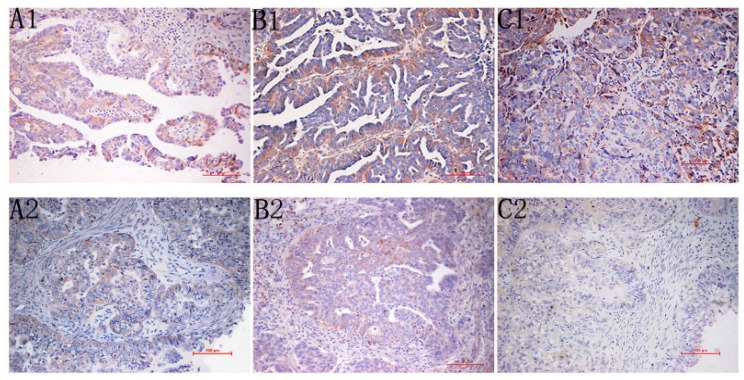
(**A**) Immunohistochemistry Staining for integrin α5 (A1; drug-resistant group, A2; drug-sensitive group, original magnification 200×); (**B**) Immunohistochemistry Staining for Integrin β1 (B1; drug-resistant group, B2; drug-sensitive group, original magnification 200×); (**C**) Immunohistochemistry Staining for Lewis y antigen, this antigen is primarily in the cell membrane and secondarily in the cytoplasm (C1; drug-resistant group, C2; drug-sensitive group, original magnification 200×).

Staining for Lewis y antigen identified this molecule primarily in the cell membrane and secondarily in the cytoplasm (Figure 1C). The positive expression rates of Lewis y antigen in the drug-resistant, partially sensitive, and sensitive groups were 91.17%, 50.00%, and 61.54%, respectively (Table 1). The positive expression rate of Lewis y in the drug-resistant group was significantly higher than in the partially sensitive group and sensitive group (0.005 < both *p* < 0.01).

Integrins α5 and β1 also were primarily localized in the cell membrane and partially in the cytoplasm (Figure 1A,B). The positive expression rates of integrin α5 in the drug-resistant, partially sensitive, and sensitive groups were 85.29%, 33.33%, and 57.69%, respectively (Table 1). The expression rate of integrin α5 in the drug-resistant group was significantly higher than in the partially sensitive (0.005 < *p* < 0.01) and sensitive groups (0.01 < *p* < 0.025). The positive expression rate of integrin β1 in the ovarian drug resistance group was 88.24%, a significant increase over the partially sensitive (50.00%) and sensitive groups (55.77%) (0.01 < both *p* < 0.025).

### 2.2. Correlations Analysis for Lewis y, Integrin α_5_, and Integrin β_1_ in Ovarian Cancer Tissues

There was a significant correlation between Lewis y and integrin α_5_ (Spearman coefficient *r_s_* = 0.5073, *p* < 0.001), and integrin β_1_ (Spearman coefficient *r_s_* = 0.4134, *p* < 0.005).

### 2.3. Univariate Analysis of Ovarian Carcinoma Chemotherapeutic Drug Resistance

Univariate analyses were conducted for pathological subtype, surgical stage, grade, metastasis of lymph nodes, and residual tumor size in the resistant and sensitive groups. Significant differences were identified in surgical stage, metastasis of lymph nodes, and residual tumor size (*p* = 0.002, 0.004, and < 0.0001, respectively) (Table 2). The other analyses failed to detect significant differences between groups (*p* > 0.05).

### 2.4. Multivariate Analysis of Ovarian Carcinoma Chemotherapeutic Drug Resistance

Age, surgical stage, grade, pathological subtype, metastasis of lymph nodes, residual tumor size after surgery, and expression levels of Lewis y antigen, integrin α_5_, and integrin β_1_ served as covariables in a binary logistic regression analysis (forward: conditional). The results revealed that surgical stage, residual tumor size, and expression of integrin α_5_ and Lewis y in ovarian carcinoma tissues were independent risk factors for chemotherapeutic drug resistance (Table 3).

### 2.5. Multivariate Analysis of Prognosis in Ovarian Carcinoma Patients

Multivariate COX model analysis of overall survival was performed for prognosis in ovarian carcinoma patients. Metastasis of lymph nodes, expression of integrin α_5_, and expression of Lewis y were independent risk factors for the prognosis of ovarian carcinoma (Table 4).

### 2.6. Comparison of Survival Rates

**Figure 3:**
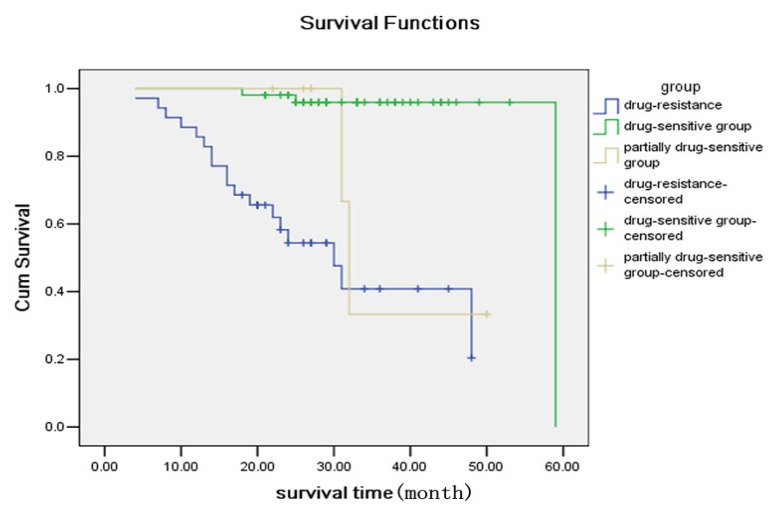
Comparison of Survival Rates.

Lewis y and integrin α_5_β_1_ were associated with ovarian carcinoma survival of the enrolled patients. The analyses as of Patients in June 2011, were over a span of six years for the earliest enrolled patients and nearly two years for the latest enrolled patients. Overall, 18 patients (52.94%) died due to metastasis and relapse of tumors in the drug-resistant group; two patients (33.33%) died in the partially drug-sensitive group; three patients (5.8%) died in the drug-sensitive group. Survival rates of the three groups of patients were evaluated via Kaplan-Meier analysis, which indicated that the mortality in the drug-resistant group and partially drug-sensitive group was significantly higher than in the drug-sensitive group. Both the log-rank test and the Breslow test returned *p* < 0.0001 (Figure 3).

## 3. Discussion

Ovarian carcinoma is associated with the highest mortality of all female genital tumors, in part, because of the resistance of ovarian carcinoma tumors to chemotherapy. The development of drug resistance involves complicated molecular mechanisms: studies investigating this phenomenon typically report decreased intracellular drug accumulation [12,13], an enhancement in DNA damage repair, or an increase in glutathione detoxification enzyme activity [14,15]. Recently, CAM-DR was the focus of several studies in this field. CAM-DR is defined as an alteration in the tumor cell adhesion components and adhesion capabilities, which results in cytoskeletal rearrangements and activates various survival signal transduction pathways. Ultimately, CAM-DR leads to enhanced inhibition of apoptosis, despite ionizing radiation and chemotherapeutic drugs.

Adhesion interactions between tumor cells and the extracellular matrix improve tumor cell survival and inhibit apoptosis. Integrins are an important family of adhesion molecules that are distributed on the cell surface and function as receptors for various extracellular components. Recently, integrins were implicated in tumor drug resistance, but detailed mechanisms explaining this relationship are unavailable. Lee *et al.*[16] found that exposure to ionizing radiation elevated the expression of β-galactoside-α(2,6) sialyltransferase and the sialylation level of integrin β_1_ in the leukemia cell line U937. This increased glycoprotein stability, enhanced anti-apoptosis capabilities, and significantly decreased the sensitivity of cells to chemotherapy. These results support the fact that adhesion molecule glycosylation on the cell surface probably affects CAM-DR. Kudo *et al.*[17] and Vagin *et al.*[18] conducted similar investigations and reported that adhesion molecule glycosylation plays a crucial role in mediating drug resistance.

Previously, we performed gene transfection to introduce the human α1,2-fucosyl-transferase gene into human ovarian clear cell carcinoma RMG-1 cells to establish the RMG-1-H cell line that highly expresses both α1,2-fucosyl-transferase and Lewis y. An increase in Lewis y antigen content on the cell surface is associated with increased expression levels of integrins α_5_β_1_ and α_v_β_3_[10,11]. Lewis y antigen is a component of the integrin α and β subunits, and Lewis y modification can affect cell adhesion, mobility, proliferation, and apoptosis [10]. We measured the expression status of Lewis y antigen and integrin α_5_β_1_ in ovarian carcinoma. Our results reveal that Lewis y antigen and integrin α_5_β_1_ are linearly correlated [19]. Compared to RMG-1 cells, RMG-1-H cells exhibit elevated expression of Lewis y antigen on the cell surface, but also exhibit enhanced proliferation and infiltration capabilities. These cells demonstrate a decreased apoptosis rate and a significantly increased resistance to various chemotherapy drugs, such as 5-Fluorouracil, carboplatin, and paclitaxel [20,21]. Exposure of cells to anti-Lewis y antibody can counteract these alterations [22].

Integrin α_5_β_1_ is a glycoprotein adhesion molecule. Glycosylation is an important post-translational modification that affects the binding strength of integrin α_5_β_1_ to its ligands and alters its roles in physiological and pathological processes, such as angiogenesis and tumor metastasis. Zhao *et al*. [23] performed α1,6-fucosyltransferase gene knockouts in mice and reported serious growth retardation and pulmonary emphysema-like effects. These knockout mice also exhibited decreased functioning of the transforming growth factor-β1 receptor and the epithelial growth factor receptor, which can interfere with cell mobility and cellular growth signals mediated by integrin α_3_β_1_. This suggests that fucosylation is required for integrin α_3_β_1_ function.

The present study used tissue samples from ovarian carcinoma patients combined with patient data, including clinical drug resistance status, to identify correlations between the expression levels of integrin α_5_β_1_ and Lewis y with ovarian carcinoma drug resistance and prognosis. The positive expression rates of integrins α_5_ and β_1_ in the drug-resistant group were significantly higher than those in the partially sensitive group or in the sensitive group (all *p* < 0.05). These results support the fact that increased integrin α_5_β_1_ expression is closely associated with ovarian carcinoma drug resistance, which is consistent with several previous studies [24,25].

Our previous studies also demonstrated that Lewis y, as part of various crucial molecules on the cell surface (e.g., integrin α_5_β_1_, α_v_β_3_, CD44, CD147), enhances cellular malignant biological behavior, such as proliferation, adhesion, metastasis, and drug resistance [6,7,26]. The current study measured positive Lewis y expression rates of 91.67%, 50.00%, and 61.54% in the drug-resistant, partially sensitive, and sensitive groups, respectively. The rate in the drug-resistant group was significantly higher than those in the partially sensitive and sensitive groups (0.005 < *p* < 0.01 for both) and there was a significant correlation between Lewis y and integrin α_5_β_1_, providing clinical support for our previous research conclusions based on *in vitro* data.

Multivariate logistic regression analyses confirmed that the surgical stage, residual tumor size, Lewis y, and integrin α_5_ were independent risk factors for chemotherapeutic drug resistance in ovarian carcinoma. In contrast, age, grade, and pathological subtype of the ovarian carcinoma patients were not correlated with chemotherapy drug resistance. In univariate analysis, metastasis of lymph nodes was correlated with chemotherapeutic drug resistance, but the correlation was not significant in multivariate analysis after interference noise was removed.

This study conducted multivariate survival analysis for 92 patients and found that integrin α_5_, Lewis y, and metastasis of lymph nodes were all risk factors for ovarian carcinoma prognosis; that is, the lymph node metastasis and higher positive expression levels of integrin α_5_ and Lewis y were associated with a worse prognosis. This result supported the fact that glycosylation plays a pivotal role in drug resistance processes of tumor cells. However, the surgical stage and residual tumor size are not correlated with the prognosis, which may be due to an insufficiently long follow-up time. Ichiro *et al.*[27] reported that tumor cell sensitivity to cisplatin significantly increases following a tunicamycin-induced blockade of the *N*-oligosaccharide chain. ATP binding cassette transporters MRP1 and MRP4 are highly expressed in the oxaliplatin-resistant ovarian carcinoma cell line, IGROV-1/OHP. *N*-chain glycosylations are abnormally increased on MRP1 and MRP4 in IGROV-1/OHP cells [28]. Prion protein PrP^c^ effects measurable antiapoptotic capabilities in oral squamous cell carcinoma HSC-2 cells and in gut adenoma LS174T cells. However, blockade of *N*-chain glycosylation by tunicamycin eliminates this antiapoptosis capability [29]. Taken together, these results are consistent with our findings.

Extensive studies have been conducted on integrin-associated tumor cell drug resistance, but no conclusions have been reached. Damiano *et al.*[5] suggest that after tumor cells adhere via integrin-mediated matrix adhesion molecules, focal adhesion kinase located in the cell membrane rapidly activates to further enhance Bcl-2 expression and inhibit apoptosis. Our prior studies demonstrate that after ovarian carcinoma cells adhere with fibronectin (FN), Bcl-2 expression is increased, and the FN-RMG-1-H group is higher than the FN-RMG-1 group. Carboplatin further increases Bcl-2 expression in RMG-1-H cells, but effects no significant change in RMG-I cells. A similar pattern was detected for Bcl-XL. Exposure to anti-Lewis y antibody decreases the expression of Bcl-2 and Bcl-XL to different extents. These results suggest that Lewis y inhibits apoptosis by regulating the expression of Bcl-2 and Bcl-XL via CAM-DR [30] Zhang *et al.*[31] report that after cellular 3′-*O*-sulfotransferase is knocked down via siRNA, sulfonated Le^x^ and integrin αv become undetectable, cell proliferation capability is reduced, and adhesion reactions with sL-selectin, fibronectin, and vitronectin all decrease. Exogenous 3-sulfonated Le^x^ causes increased cell-surface expression of integrin α_v_β_3_, elevated expression of phosphorylated AKT and ERK, and decreased Bcl-2 with a decreased Bcl/Bax ratio. These results are in accordance with our findings.

Saccharide chains on the cell membrane function like antennae as important mediators of information between cells and between cells and their external environments. Studies suggest that alterations in the quality and quantity of surface glycoproteins can dismantle signaling cascades, which further lead to enhanced activities in NF-κB antiapoptosis signal pathways and PI3K/AKT survival signal pathways and reduce the activity as well as the nuclear redistribution of topoisomerase II. Cumulatively, these activity changes result in increased drug resistance of ovarian carcinoma cells [32–34].

Our prior work supports that Lewis y, a component of various cell-surface receptors, promotes a variety of biological alterations, including cell adhesion and metastasis, by activating ERK/MAPK and PI3K/AKT signal transduction pathways. In addition, Lewis y inhibits apoptosis and induces drug resistance via a P38 MAPK pathway, thereby suggesting a pro-survival role for such a pathway. Exposure to an anti-Lewis y antibody can inhibit these changes [35–37]. The current study used drug-resistant clinical ovarian carcinoma samples to demonstrate that Lewis y and integrin α_5_β_1_ were associated with ovarian carcinoma drug resistance. We report that Lewis y and integrin α5 are independent risk factors associated with ovarian carcinoma drug resistance and with poorer prognosis. In light of our previous studies, we suggest that Lewis y is a key molecule in CAM-DR with potential as a novel antiapoptosis target for the treatment of drug resistance in ovarian carcinoma.

## 4. Materials and Methods

### 4.1. Materials

Between May 2005 and July 2009, 92 Chinese patients diagnosed with primary epithelial ovarian cancer by surgery and pathological analyses were retrospectively assembled from the China Medical University Hospital. Patients had undergone cytoreductive surgery and systematic chemotherapy based on paclitaxel and carboplatin for 6–8 standard periods, the beginning time of chemotherapy follow-up was designated on the day of the patients’ surgery, and the follow-up visiting lasted for nearly two years. We collected the clinical and chemotherapy follow-up information of patients with approval from the Institutional Review Board of Shengjing Hospital. All the clinical and tumor information is presented in Table 2.

According to NCCN (National Comprehensive Cancer Network) guidelines we divided all patients into an ovarian cancer chemotherapy drug-resistant group (34 cases), a partially sensitive group (six cases) and a sensitive group (52 cases). They met the requirements as follows: patients in the drug-resistant group firstly adopted chemotherapy based on paclitaxel and carboplatin and gained alleviation, but was followed by recurrence in the six months after chemotherapy; patients in the partial sensitive group relapsed 6 months to 12 months after chemotherapy; patients in the sensitive group achieved alleviation for at least 12 months. The main signs of recurrence of ovarian cancer include: a constant increase in CA125; detection of abdominal mass in gynecologial examination; detection of abdominal mass in imaging findings as well as signs of ascites.

### 4.2. Immunohistochemistry

The paraffin embedding histological section of each group of ovarian tissue was 5 μm. Immunohistochemistry was used to analyze Lewis y antigen and integrin α_5_, β_1_. Mouse monoclonal anti-Lewis y antibody (clone A70-C/C8) was bought from Abcam Company (Cambridge, UK), and rabbit polyclonal anti-α_5_ and anti-β_1_antibodies were bought from Boshide Biotech (Wuhan, China). The working concentrations of Lewis y antibody and integrin α_5_, β_1_ antibody were 1:160, 1:200 and 1:300, respectively. The staining procedure was performed according to SP (streptavidin-perosidase) kit manual (kit-9701, Maixin.Bio, Fuzhou, China), and using Harris’s hematoxylin for nuclei. The group with PBS instead of primary antibody was used as a negative control. A colon cancer tissue sample and a breast cancer sample served as positive control for Lewis y antigen, and for integrin α_5_, β_1_ respectively.

We considered a positive result if there were buff colored granules in the cell membrane and cytoplasm. According to the chromatosis intensity, no pigmentation, light yellow, buff, or brown are scored 0,1,2,3, respectively. We chose five high-power fields in series from each slice, then scored them and took the mean percentage of the chromatosis cells: chromatosis cells with a count less than 5% are zero; 5% to 25%, one; 26% to 50%, two; 51% to 75%, three; and greater than 75%, four. Multiply these two numbers; 0 to 2 is considered (-); 3 to 4, (+); 5 to 8, (++), and 9 to 12, (+++). Score ≥ 3 tumours are considered positive for Lewis y antigen and integrin α_5_, β_1_ in statistical analysis. Two observers read the sections to control the error.

### 4.3. Statistical Analysis

Positive ratio rates were evaluated using the *X^2^* test, correlation analyses were performed using Spearman’s rank order correlation coefficient. Binary logistic regression was used for multivariate analysis. The Cox proportional hazard regression model was used to indentify independent prognostic factors for overall survival, with variables being entered in a single step. All statistical analyses were performed using SPSS V13.0 software (SPSS, Chicago, IL, USA). A two-tailed *p* value test was used in all analyses; *p*-values less than 0.05 were considered statistically significant.

## 5. Conclusions

We measured the expression levels of Lewis y antigen and integrin α_5_β_1_, finding that there was a significant correlation between Lewis y and integrin α_5_ and integrin β_1_. On evaluating their localization and correlation in ovarian carcinoma tissues, obtained from drug-resistant and non-drug-resistant patients, we found overexpression of Lewis y and integrin α_5_ are strong risk factors for chemotherapeutic drug resistance and prognosis of ovarian carcinoma.

## Figures and Tables

**Table 1:** The expression of Lewis y and integrin α5β1 in ovarian cancer tissues.

	Group	Drug-resistant	Partial sensitive	Sensitive group
Lewis y antigen	case	34	6	52
	−	3	3	20
	+	4	2	14
	++	19	1	18
	+++	8	0	0
	* Positive ratio (%)	91.17 (31/34)	50.00 (3/6)	61.54 (32/52)
integrinα5	−	5	4	22
	+	3	1	12
	++	23	1	18
	+++	3	0	0
	* Positive ratio (%)	85.29 (29/34)	33.33 (2/6)	57.69 (30/2)
tegrinβ1	−	4	3 23	
	+	9	2	15
	++	18	1	13
	+++	3	0	1
	* Positive ratio (%)	88.24 (30/34)	50.00 (3/6)	55.77 (29/52)

* Score ≥ 3(+) tumours are considered positive for Lewis y antigen and integrin α_5_, β_1_ in statistical analysis.

**Table 2:** Univariate analyses of ovarian carcinoma chemotherapeutic drug resistance.

Classification	Total	Drug-resistant		Sensitive group #		*F*-value	*p*-Value
	
		Case	Percentage	Case	Percentage		
Surgical stage							
I~II stage	32	4	12.50%	28	87.50%	5.359	0.002
III~IV stage	60	30	50.00%	30	50.00%		
Tumor grade							
I grade	15	4	26.67%	11	73.33%		
II grade	37	10	27.03%	27	72.97%	3.033	0.053
III grade	40	20	50.00%	20	50.00%		
Histotype							
Serous carcinoma	58	24	41.38%	34	58.62%	0.776	0.544
Mucinous carcinoma	8	4	50.00%	4	50.00%		
Endometrioid adenocarcinoma	4	1	25.00%	3	75.00%		
Clear cell carcinoma	6	1	16.67%	5	83.33%		
Poorly differentiated carcinoma	16	5	31.25%	11	68.75%		
metastasis of lymph node	60 *						
yes	13	8	61.54%	5	38.46%		
no	47	9	19.15%	38	80.85%	7.599	0.004
residual tumor size	71 **						
≤1 cm	43	6	13.95%	37	86.05%		
1~2 cm	16	7	43.75%	9	56.25%	9.927	0.000
≥2 cm	12	10	83.33%	2	16.67%		

* 60 cases had lymph node data;

** 71 cases had residual tumor size data;

# partially sensitive patients were grouped with sensitive patients.

**Table 3:** Multivariate analysis of ovarian carcinoma chemotherapeutic drug resistance.

Type	*p*-Value	Hazard ratio (95% CI)
Stage	0.035	2.556 (1.067~6.123)
Residual tumor size	0.004	1.721 (1.185~2.498)
Integrinα5	0.005	4.030 (1.975~8.225)
Lewis y	0.016	2.154 (1.156~4.014)

**Table 4:** Multivariate analysis of prognosis in ovarian carcinoma patients.

Type	*p*-Value	Hazard ratio (95% CI)
Metastasis of lymph node	0.016	1.832 (1.119~3.000)
Integrin α_5_	0.019	1.876 (1.110~3.170)
Lewis y antigen	0.037	1.911 (1.136~4.323)
